# Supplementary material for: An assessment of vaccine wastage in the Solomon Islands
Source: PLOS Glob Public Health. 2022 Jun 9;2(6):e0000572. doi: 10.1371/journal.pgph.0000572 (PMC10021283; doi:10.1371/journal.pgph.0000572)
Supplement: S1 Text — (DOCX) [file pgph.0000572.s001.docx]

*Table A*


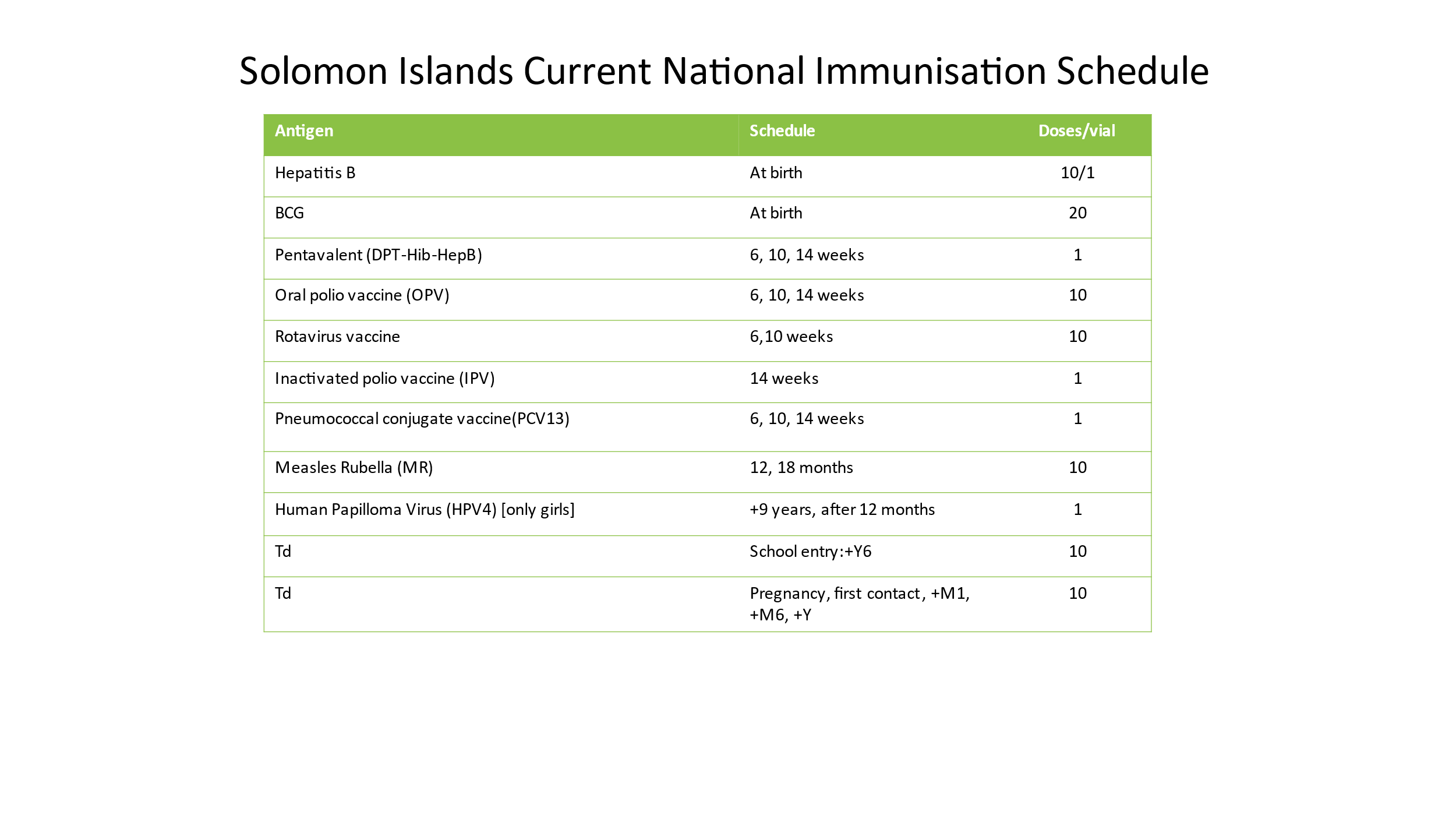
*Table B Solomon Islands: types of health facilities and total number selected by province*

|  | | **Total number of facilities by type** | | | | | | **Total no selected** |
| --- | --- | --- | --- | --- | --- | --- | --- | --- |
| **Province** | **Population** | **Total** | **AHC** | **NAP** | **Hospital** | **Mobile clinic** | **RHC** |  |
| *Selected* |  |  |  |  |  |  |  |  |
| Guadalcanal | 144,592 | 44 | 6 | 24 | 0 | 0 | 14 | 4 |
| Honiara | 86,529 | 22 | 4 | 10 | 1* | 2 | 5 | 3 |
| Malaita | 158,076 | 78 | 4 | 52 | 2 | 0 | 20 | 6 |
| Renbel | 4,026 | 3 | 1 | 0 | 0 | 0 | 2 | 2 |
| Temotu | 24,757 | 16 | 2 | 8 | 1 | 0 | 5 | 2 |
| Western | 95,579 | 58 | 6 | 27 | 2 | 0 | 23 | 5 |
|  |  |  |  |  |  |  |  |  |
| *Others* |  |  |  |  |  |  |  |  |
| Central Islands | 31,732 | 23 | 4 | 14 | 1 | 0 | 0 | - |
| Choiseul | 35,035 | 26 | 2 | 13 | 1 | 0 | 0 | - |
| Isabel | 33,843 | 33 | 5 | 17 | 1 | 0 | 0 | - |
| Makira | 52,880 | 40 | 4 | 19 | 1 | 0 | 0 | - |
|  | **607,044** | **343** | **38** | **184** | **10** | **2** | **109** | **22** |
|  | | *national referral Hospital | | | | | | |

*Table C Wastage rate for* ***multidose vials*** *(1) SLMS and (2) all other facilities*

*(Based on wasted/discarded doses at the selected facilities)*

1. SLMs

| **Vaccine** | **Facility** | **Year** | **Wastage** | **Doses discarded** | **Immunised** | **Usage** |
| --- | --- | --- | --- | --- | --- | --- |
| BCG | **401** | 2017 |  | nr | 33 |  |
|  |  | 2018 |  | nr | 62 |  |
|  | **405** | 2017 |  | nr | 715 |  |
|  |  | 2018 | 0.0 | 0 | 716 | 100.0 |
|  | **601** | 2017 | 95.7 | 264 | 12 | 4.3 |
|  |  | 2018 | 91.0 | 122 | 12 | 9.0 |
|  | **702** | 2017 |  | nr | 203 |  |
|  |  | 2018 | 0.0 | 0 | 228 | 100.0 |
|  | **706** | 2017 | 0.0 | 0 | 720 | 100.0 |
|  |  | 2018 | 0.0 | 0 | 944 | 100.0 |
|  | **801** | 2017 |  | nr | 13 |  |
|  |  | 2018 |  | nr | 13 |  |
|  | **902** | 2017 | 100.0 | 206 | 0 | 0.0 |
|  |  | 2018 | 100.0 | 84 | 0 | 0.0 |
|  |  |  |  |  |  |  |
| MR | **401** | 2017 |  | nr | 43 |  |
|  |  | 2018 |  | nr | 258 |  |
|  | **405** | 2017 |  | nr | 0 |  |
|  |  | 2018 |  | 0 | 0 |  |
|  | **601** | 2017 | 0.0 | 0 | 468 | 100.0 |
|  |  | 2018 | 4.9 | 23 | 444 | 95.1 |
|  | **702** | 2017 |  | nr | 179 |  |
|  |  | 2018 | 0.0 | 0 | 319 | 100.0 |
|  |  | 2018 |  | nr | 37 |  |
|  | **705** | 2017 |  | nr | 126 |  |
|  |  | 2018 |  | nr | 99 |  |
|  | **801** | 2017 |  | nr | 22 |  |
|  |  | 2018 |  | nr | 91 |  |
|  | **902** | 2017 |  | 0 | 0 |  |
|  |  | 2018 |  | nr | 0 |  |
|  |  |  |  |  |  |  |
| TT | **401** | 2017 |  | nr | 40 |  |
|  |  | 2018 |  | nr | 389 |  |
|  | **405** | 2017 |  | nr | 69 |  |
|  |  | 2018 | 0.0 | 0 | 113 | 100.0 |
|  | **601** | 2017 | 0.0 | 0 | 247 | 100.0 |
|  |  | 2018 | 0.0 | 0 | 738 | 100.0 |
|  | **702** | 2017 |  | nr | 616 |  |
|  |  | 2018 | 0.0 | 0 | 491 | 100.0 |
|  | **706** | 2017 | 0.0 | 0 | 108 | 100.0 |
|  |  | 2018 | 0.0 | 0 | 102 | 100.0 |
|  | **801** | 2017 |  | nr | 17 |  |
|  |  | 2018 |  | nr | 134 |  |
|  | **902** | 2017 | 0.0 | 0 | 117 | 100.0 |
|  |  | 2018 |  | nr | 127 |  |
|  |  |  |  |  |  |  |
| OPV | **401** | 2017 |  | nr | 15 |  |
|  |  | 2018 |  | nr | 720 |  |
|  | **405** | 2017 |  | nr | 0 |  |
|  |  | 2018 |  | 0 | 0 |  |
|  | **601** | 2017 | 100.0 | 154 | 0 | 0.0 |
|  |  | 2018 | 0.0 | 0 | 1250 | 100.0 |
|  | **702** | 2017 |  | nr | 424 |  |
|  |  | 2018 | 0.0 | 0 | 544 | 100.0 |
|  | **706** | 2017 |  | 0 | 0 |  |
|  |  | 2018 |  | 0 | 0 |  |
|  | **801** | 2017 |  | nr | 0 |  |
|  |  | 2018 |  | nr | 203 |  |
|  | **902** | 2017 | 100.0 | 1 | 0 | 0.0 |
|  |  | 2018 |  | 0 | 0 |  |
|  |  |  |  |  |  |  |
| HepB | **401** | 2017 |  | nr | 43 |  |
|  |  | 2018 |  | nr | 52 |  |
|  | **405** | 2017 |  | nr | 733 |  |
|  |  | 2018 | 0.0 | 0 | 729 | 100.0 |
|  | **601** | 2017 | 0.0 | 0 | 1 | 100.0 |
|  |  | 2018 | 62.5 | 10 | 6 | 37.5 |
|  | **702** | 2017 |  | nr | 197 |  |
|  |  | 2018 | 0.0 | 0 | 161 | 100.0 |
|  | **706** | 2017 | 25.3 | 300 | 885 | 74.7 |
|  |  | 2018 | 0.0 | 0 | 947 | 100.0 |
|  | **801** | 2017 |  | nr | 8 |  |
|  |  | 2018 |  | nr | 13 |  |
|  | **902** | 2017 | 100.0 | 5 | 0 | 0.0 |
|  |  | 2018 |  | 0 | 0 |  |
| HepS | **902** | 2018 |  | 0 |  |  |

1. Other facilities

| **Vaccine** | **Facility** | **Year** | **Wastage** | **Doses discarded** | **immunised** | **Usage** |
| --- | --- | --- | --- | --- | --- | --- |
| BCG | 402 | 2017 |  | nr | 11 |  |
|  |  | 2018 | 0.0 | 0 | 26 | 100.0 |
|  | 403 | 2017 |  | nr | 8 |  |
|  |  | 2018 |  | nr | 19 |  |
|  | 404 | 2017 | 0.0 | 0 | 6 | 100.0 |
|  |  | 2018 | 0.0 | 0 | 5 | 100.0 |
|  | 501 | 2017 | 63.2 | 115 | 67 | 36.8 |
|  |  | 2018 |  | nr | 57 |  |
|  | 502 | 2017 | 0.0 | 0 | 23 | 100.0 |
|  |  | 2018 | 66.7 | 14 | 7 | 33.3 |
|  | 503 | 2017 |  | nr | 7 |  |
|  |  | 2018 |  | nr | 0 |  |
|  | 504 | 2017 | 0.0 | 0 | 643 | 100.0 |
|  |  | 2018 | 0.0 | 0 | 657 | 100.0 |
|  | 602 | 2017 |  | nr | 1 |  |
|  |  | 2018 |  | nr | 0 |  |
|  | 603 | 2017 |  | 0 | 0 |  |
|  |  | 2018 | 100.0 | 27 | 0 | 0.0 |
|  | 701 | 2017 | 88.6 | 404 | 52 | 11.4 |
|  |  | 2018 | 33.7 | 32 | 63 | 66.3 |
|  | 703 | 2017 |  | nr | 56 |  |
|  |  | 2018 |  | nr | 21 |  |
|  | 704 | 2017 |  | nr | 7 |  |
|  |  | 2018 |  | nr | 2 |  |
|  | 705 | 2017 |  | nr | 117 |  |
|  |  | 2018 |  | nr | 78 |  |
|  | 802 | 2017 |  | nr | 2 |  |
|  |  | 2018 |  | nr | 1 |  |
|  | 901 | 2017 |  | nr | 1 |  |
|  |  | 2018 |  | nr | 0 |  |
|  |  |  |  |  |  |  |
| MR | 402 | 2017 |  | nr | 32 |  |
|  |  | 2018 |  | nr | 96 |  |
|  | 403 | 2017 |  | nr | 25 |  |
|  |  | 2018 |  | nr | 122 |  |
|  | 404 | 2017 |  | nr | 17 |  |
|  |  | 2018 | 0.0 | 0 | 17 | 100.0 |
|  | 501 | 2017 | 47.6 | 81 | 89 | 52.4 |
|  |  | 2018 |  | nr | 66 |  |
|  | 502 | 2017 |  | nr | 42 |  |
|  |  | 2018 | 86.6 | 414 | 64 | 13.4 |
|  | 503 | 2017 |  | nr | 15 |  |
|  |  | 2018 |  | nr | 2 |  |
|  | 504 | 2017 | 0.0 | 0 | 523 | 100.0 |
|  |  | 2018 | 0.0 | 0 | 496 | 100.0 |
|  | 602 | 2017 |  | nr | 57 |  |
|  |  | 2018 |  | nr | 48 |  |
|  | 603 | 2017 |  | nr | 29 |  |
|  |  | 2018 | 43.8 | 142 | 182 | 56.2 |
|  | 701 | 2017 | 39.8 | 41 | 62 | 60.2 |
|  |  | 2018 | 2.0 | 15 | 717 | 98.0 |
|  | 703 | 2017 |  | nr | 75 |  |
|  |  | 2018 |  | nr | 90 |  |
|  | 704 | 2017 |  | nr | 12 |  |
|  |  | 2018 |  | nr | 37 |  |
|  | 706 | 2017 |  | 0 | 0 |  |
|  |  | 2018 |  | 0 | 0 |  |
|  | 802 | 2017 |  | nr | 21 |  |
|  |  | 2018 |  | nr | 8 |  |
|  | 901 | 2017 |  | nr | 2 |  |
|  |  | 2018 |  | nr | 3 |  |
|  |  |  |  |  |  |  |
| TT | 402 | 2017 |  | nr | 0 |  |
|  |  | 2018 |  | nr | 24 |  |
|  | 403 | 2017 |  | nr | 0 |  |
|  |  | 2018 |  | nr | 83 |  |
|  | 404 | 2017 |  | nr | 4 |  |
|  |  | 2018 |  | nr | 37 |  |
|  | 501 | 2017 | 0.0 | 0 | 119 | 100.0 |
|  |  | 2018 | 0.0 | 0 | 234 | 100.0 |
|  | 502 | 2017 | 0.0 | 0 | 57 | 100.0 |
|  |  | 2018 | 14.6 | 14 | 82 | 85.4 |
|  | 503 | 2017 |  | nr | 3 |  |
|  |  | 2018 |  | nr | 11 |  |
|  | 504 | 2017 | 0.0 | 0 | 145 | 100.0 |
|  |  | 2018 | 0.0 | 0 | 1290 | 100.0 |
|  | 602 | 2017 |  | nr | 24 |  |
|  |  | 2018 |  | nr | 195 |  |
|  | 603 | 2017 | 0.0 | 0 | 990 | 100.0 |
|  |  | 2018 | 27.6 | 352 | 922 | 72.4 |
|  | 701 | 2017 | 0.0 | 0 | 148 | 100.0 |
|  |  | 2018 | 0.0 | 0 | 238 | 100.0 |
|  | 703 | 2017 |  | nr | 29 |  |
|  |  | 2018 |  | nr | 118 |  |
|  | 704 | 2017 |  | nr | 7 |  |
|  |  | 2018 |  | nr | 5 |  |
|  | 705 | 2017 |  | nr | 358 |  |
|  |  | 2018 |  | nr | 295 |  |
|  | 802 | 2017 |  | nr | 27 |  |
|  |  | 2018 |  | nr | 20 |  |
|  | 901 | 2017 |  | nr | 0 |  |
|  |  | 2018 |  | nr | 0 |  |
|  |  |  |  |  |  |  |
| OPV | 402 | 2017 |  | nr | 0 |  |
|  |  | 2018 |  | nr | 169 |  |
|  | 403 | 2017 |  | nr | 0 |  |
|  |  | 2018 |  | nr | 133 |  |
|  | 404 | 2017 |  | 0 | 0 |  |
|  |  | 2018 | 0.0 | 0 | 63 | 100.0 |
|  | 501 | 2017 | 100.0 | 158 | 0 | 0.0 |
|  |  | 2018 | 0.0 | 0 | 187 | 100.0 |
|  | 502 | 2017 |  | 0 | 0 |  |
|  |  | 2018 | 0.0 | 0 | 93 | 100.0 |
|  | 503 | 2017 |  | nr | 0 |  |
|  |  | 2018 |  | nr | 1 |  |
|  | 504 | 2017 | 0.0 | 0 | 51 | 100.0 |
|  |  | 2018 | 0.0 | 0 | 1670 | 100.0 |
|  | 602 | 2017 |  | nr | 0 |  |
|  |  | 2018 |  | nr | 212 |  |
|  | 603 | 2017 |  | nr | 655 |  |
|  |  | 2018 | 44.3 | 808 | 1017 | 55.7 |
|  | 701 | 2017 | 92.1 | 303 | 26 | 7.9 |
|  |  | 2018 | 0.0 | 0 | 595 | 100.0 |
|  | 703 | 2017 |  | nr | 170 |  |
|  |  | 2018 |  | nr | 232 |  |
|  |  | 2017 |  | nr | 12 |  |
|  | 704 | 2018 |  | nr | 13 |  |
|  | 705 | 2017 |  | nr | 56 |  |
|  |  | 2018 |  | nr | 328 |  |
|  | 802 | 2017 |  | nr | 5 |  |
|  |  | 2018 |  | nr | 16 |  |
|  | 901 | 2017 |  | nr | 0 |  |
|  |  | 2018 |  | nr | 0 |  |
|  |  |  |  |  |  |  |
| HepB | 402 | 2017 |  | nr | 7 |  |
|  |  | 2018 |  | nr | 14 |  |
|  | 403 | 2017 |  | nr | 9 |  |
|  |  | 2018 |  | nr | 15 |  |
|  | 404 | 2017 | 0.0 | 0 | 6 | 100.0 |
|  |  | 2018 | 0.0 | 0 | 11 | 100.0 |
|  | 501 | 2017 | 47.9 | 46 | 50 | 52.1 |
|  |  | 2018 | 38.1 | 37 | 60 | 61.9 |
|  | 502 | 2017 | 0.0 | 0 | 1 | 100.0 |
|  |  | 2018 | 71.4 | 5 | 2 | 28.6 |
|  | 503 | 2017 |  | nr | 0 |  |
|  |  | 2018 |  | nr | 0 |  |
|  | 504 | 2017 | 0.0 | 0 | 595 | 100.0 |
|  |  | 2018 | 0.0 | 0 | 663 | 100.0 |
|  | 602 | 2017 |  | nr | 0 |  |
|  |  | 2018 |  | nr | 0 |  |
|  | 603 | 2017 |  | 0 | 0 |  |
|  |  | 2018 |  | 0 | 0 |  |
|  | 701 | 2017 | 22.9 | 19 | 64 | 77.1 |
|  |  | 2018 | 0.0 | 0 | 49 | 100.0 |
|  | 703 | 2017 |  | nr | 18 |  |
|  |  | 2018 |  | nr | 6 |  |
|  | 704 | 2017 |  | nr | 3 |  |
|  |  | 2018 |  | nr | 1 |  |
|  | 705 | 2017 |  | nr | 101 |  |
|  |  | 2018 |  | nr | 78 |  |
|  | 802 | 2017 |  | nr | 0 |  |
|  |  | 2018 |  | nr | 0 |  |
|  | 901 | 2017 |  | nr | 0 |  |
|  |  | 2018 |  | nr | 0 |  |

*Table D Wastage rates for* ***single dose vials*** *(1) SLMS and (2) all other facilities*

*(based on wasted/discarded doses at facilities)*

(1) SLMS

| **Vaccine** | **Facility** | **Year** | **Wastage** | **Doses discarded** | **immunised** | **Usage** |
| --- | --- | --- | --- | --- | --- | --- |
| IPV | **401** | 2017 |  | nr | 65 |  |
|  |  | 2018 |  | nr | 93 |  |
|  | **405** | 2017 |  | nr | 0 |  |
|  |  | 2018 |  | 0 | 0 |  |
|  | **601** | 2017 | 0.0 | 0 | 451 | 100.0 |
|  |  | 2018 | 0.0 | 0 | 351 | 100.0 |
|  | **702** | 2017 |  | nr | 182 |  |
|  |  | 2018 | 0.0 | 0 | 146 | 100.0 |
|  | **706** | 2017 |  | 0 | 0 |  |
|  |  | 2018 |  | 0 | 0 |  |
|  | **801** | 2017 |  | nr | 31 |  |
|  |  | 2018 |  | nr | 55 |  |
|  | **902** | 2017 |  | 0 | 0 |  |
|  |  | 2018 | 100.0 | 4 | 0 | 0.0 |
|  |  |  |  |  |  |  |
| PCV | **401** | 2017 |  | nr | 0 |  |
|  |  | 2018 |  | nr | 305 |  |
|  | **405** | 2017 |  | nr | 0 |  |
|  |  | 2018 |  | 0 | 0 |  |
|  |  | 2018 | 0.0 | 0 | 1198 | 100.0 |
|  | **601** | 2017 |  | 0 | 0 |  |
|  |  | 2018 | 0.0 | 0 | 1042 | 100.0 |
|  | **702** | 2017 |  | nr | 0 |  |
|  |  | 2018 | 31.7 | 220 | 475 | 68.3 |
|  | **706** | 2017 |  | 0 | 0 |  |
|  |  | 2018 |  | 0 | 0 |  |
|  | **801** | 2017 |  | nr | 0 |  |
|  |  | 2018 |  | nr | 143 |  |
|  | **902** | 2017 |  | nr | 0 |  |
|  |  | 2018 |  | 0 | 0 |  |
|  |  |  |  |  |  |  |
| Penta | **401** | 2017 |  | nr | 0 |  |
|  |  | 2018 |  | nr | 283 |  |
|  | **405** | 2017 |  | nr | 0 |  |
|  |  | 2018 |  | 0 | 0 |  |
|  | **601** | 2017 |  | 0 | 0 |  |
|  |  | 2018 | 0.0 | 0 | 1026 | 100.0 |
|  | **702** | 2017 |  | nr | 0 |  |
|  |  | 2018 | 0.0 | 0 | 475 | 100.0 |
|  | **706** | 2017 |  | 0 | 0 |  |
|  |  | 2018 |  | 0 | 0 |  |
|  | **801** | 2017 |  | nr | 0 |  |
|  |  | 2018 |  | nr | 140 |  |
|  | **902** | 2017 |  | 0 | 0 |  |
|  |  | 2018 | 100.0 | 9 | 0 | 0.0 |

(2) Other facilities

| **Vaccine** | **Facility** | **Year** | **Wastage** | **Doses discarded** | **immunised** | **Usage** |
| --- | --- | --- | --- | --- | --- | --- |
| IPV | 402 | 2017 |  | nr | 33 |  |
|  |  | 2018 |  | nr | 51 |  |
|  | 403 | 2017 |  | nr | 15 |  |
|  |  | 2018 |  | nr | 31 |  |
|  | 404 | 2017 | 0.0 | 0 | 7 | 100.0 |
|  |  | 2018 | 0.0 | 0 | 5 | 100.0 |
|  | 501 | 2017 | 12.4 | 12 | 85 | 87.6 |
|  |  | 2018 |  | nr | 59 |  |
|  | 502 | 2017 |  | nr | 41 |  |
|  |  | 2018 | 50.0 | 21 | 21 | 50.0 |
|  | 503 | 2017 |  | nr | 3 |  |
|  |  | 2018 |  | nr | 0 |  |
|  | 504 | 2017 | 0.0 | 0 | 470 | 100.0 |
|  |  | 2018 | 0.0 | 0 | 469 | 100.0 |
|  | 602 | 2017 |  | nr | 77 |  |
|  |  | 2018 |  | nr | 68 |  |
|  | 603 | 2017 |  | nr | 1 |  |
|  |  | 2018 | 0.0 | 0 | 6 | 100.0 |
|  | 701 | 2017 | 55.6 | 65 | 52 | 44.4 |
|  |  | 2018 | 0.0 | 0 | 83 | 100.0 |
|  | 703 | 2017 |  | nr | 50 |  |
|  |  | 2018 |  | nr | 58 |  |
|  | 704 | 2017 |  | nr | 11 |  |
|  |  | 2018 |  | nr | 1 |  |
|  | 705 | 2017 |  | nr | 79 |  |
|  |  | 2018 |  | nr | 103 |  |
|  |  | 2018 |  | nr | 55 |  |
|  | 802 | 2017 |  | nr | 14 |  |
|  |  | 2018 |  | nr | 7 |  |
|  | 901 | 2017 |  | nr | 0 |  |
|  |  | 2018 |  | nr | 0 |  |
|  |  |  |  |  |  |  |
| PCV | 402 | 2017 |  | nr | 0 |  |
|  |  | 2018 |  | nr | 171 |  |
|  | 403 | 2017 |  | nr | 0 |  |
|  |  | 2018 |  | nr | 91 |  |
|  | 404 | 2017 |  | 0 | 0 |  |
|  |  | 2018 | 0.0 | 0 | 30 | 100.0 |
|  | 501 | 2017 |  | 0 | 0 |  |
|  |  | 2018 | 0.0 | 0 | 182 | 100.0 |
|  | 502 | 2017 |  | nr | 0 |  |
|  |  | 2018 |  | nr | 63 |  |
|  | 503 | 2017 |  | nr | 0 |  |
|  |  | 2018 |  | nr | 5 |  |
|  | 504 | 2017 |  | 0 | 0 |  |
|  |  | 2018 | 0.0 | 0 | 1198 | 100.0 |
|  | 602 | 2017 |  | nr | 0 |  |
|  |  | 2018 |  | nr | 214 |  |
|  | 603 | 2017 |  | 0 | 0 |  |
|  |  | 2018 | 0.0 | 0 | 20 | 100.0 |
|  | 701 | 2017 |  | 0 | 0 |  |
|  |  | 2018 | 0.0 | 0 | 341 | 100.0 |
|  | 703 | 2017 |  | nr | 150 |  |
|  |  | 2018 |  | nr | 231 |  |
|  | 704 | 2017 |  | nr | 0 |  |
|  |  | 2018 |  | nr | 11 |  |
|  | 705 | 2017 |  | nr | 0 |  |
|  |  | 2018 |  | nr | 311 |  |
|  | 802 | 2017 |  | nr | 0 |  |
|  |  | 2018 |  | nr | 13 |  |
|  | 901 | 2017 |  | nr | 9 |  |
|  |  | 2018 |  | nr | 0 |  |
|  |  |  |  |  |  |  |
| Penta | 402 | 2017 |  | nr | 0 |  |
|  |  | 2018 |  | nr | 159 |  |
|  | 403 | 2017 |  | nr | 0 |  |
|  |  | 2018 |  | nr | 89 |  |
|  | 404 | 2017 |  | 0 | 0 |  |
|  |  | 2018 | 0.0 | 0 | 28 | 100.0 |
|  | 501 | 2017 |  | 0 | 0 |  |
|  |  | 2018 | 0.0 | 0 | 187 | 100.0 |
|  | 502 | 2017 |  | nr | 0 |  |
|  |  | 2018 |  | nr | 70 |  |
|  | 503 | 2017 |  | nr | 0 |  |
|  |  | 2018 |  | nr | 7 |  |
|  | 504 | 2017 |  | 0 | 0 |  |
|  |  | 2018 | 0.0 | 0 | 1200 | 100.0 |
|  | 602 | 2017 |  | nr | 0 |  |
|  |  | 2018 |  | nr | 202 |  |
|  | 603 | 2017 |  | 0 | 0 |  |
|  |  | 2018 | 64.8 | 35 | 19 | 35.2 |
|  | 701 | 2017 |  | 0 | 0 |  |
|  |  | 2018 | 0.0 | 0 | 313 | 100.0 |
|  | 703 | 2017 |  | nr | 149 |  |
|  |  | 2018 |  | nr | 230 |  |
|  | 704 | 2017 |  | nr | 0 |  |
|  |  | 2018 |  | nr | 10 |  |
|  | 705 | 2017 |  | nr | 0 |  |
|  |  | 2018 |  | nr | 302 |  |
|  |  | 2018 |  | nr | 140 |  |
|  | 802 | 2017 |  | nr | 0 |  |
|  |  | 2018 |  | nr | 13 |  |
|  | 901 | 2017 |  | nr | 9 |  |
|  |  | 2018 |  | nr | 0 |  |

Nr- no records

*Table E Type of wastage at the facilities (data from vaccine stock control books)*

| **Facility** | **Province** | **Vaccine** | **Remarks** |  | |  | |
| --- | --- | --- | --- | --- | --- | --- | --- |
| **Damaged** |  |  |  | | |  | |
| 702 | Malaita | PCV | 360 Vials Damaged | | |  | |
| 502 | Guadalcanal | BCG | 10 damage |  | |  | |
| 502 | Guadalcanal | Hep B | 1 damaged |  | |  | |
| 502 | Guadalcanal | IPV | 21 damaged |  | |  | |
| 502 | Guadalcanal | MR | 410 damaged |  | |  | |
|  |  |  |  |  | |  | |
| **Expiry** |  |  |  |  | |  | |
| 601^a^ | HCC | OPV | All OPV Vials Expired - Returned to VDC | | | | |
| 701 | Malaita | IPV | 65 expired which were discarded | | | | |
| 706^a^ | Malaita | HepB | 30 Vials expired 01/05/2017 | | | | |
| 902 | Temotu | Hep B | 5 vials expire so return to SLMS | | | | |
|  |  |  |  |  | |  | |
| **Power supply^b^** | |  |  |  | |  | |
| 601^a^ | HCC | BCG | Refrigerator was not function well | | | | |
| 601^a^ | HCC | PENTA | POWER OFF |  | |  | |
| 603 | HCC | PCV | Vaccine Discarded due to Power Outage | | | | |
| 601^a^ | HCC | PCV | P/off |  | |  | |
| 603 | HCC | Penta | vaccines discarded because of power outage. | | | | |
|  |  |  |  | | | | |
| **VVM 3/4** |  |  |  | | | | |
| 603 | HCC | OPV | 380 doses VVM3/4 | | | | |
|  |  |  |  |  | |  | |
| **Wastage doses^c^** | | | |  | |  | |
| 601^a^ | HCC | OPV | Wastage | |  | |  |
| 601^a^ | HCC | MR | 315 doses wastage (total) | | | |  |
| 601^a^ | HCC | Penta | 1 dose wastage / 7 Vials left | |  | |  |
| 601^a^ | HCC | BCG | Wastage | |  | |  |
| 601^a^ | HCC | BCG | Wastage | |  | |  |
| 601^a^ | HCC | BCG | Wastage | |  | |  |
| 601^a^ | HCC | BCG | 9 doses wastage | | | |  |
| 701 | Malaita | Hep B | 19 doses wastage | | | |  |
| 702 | Malaita | OPV | 157 doses wastage (total) | | | |  |
| 504 | Guadalcanal | BCG | Discarded | |  | |  |

^a^SLMS, ^b^ doses for wastage due to power issues were not stated

^c^ type not specified - all but one was mutidose vial vaccines mostly due to doses not administered
