# Supplementary material for: An assessment of vaccine wastage in the Solomon Islands
Source: PLOS Glob Public Health. 2022 Jun 9;2(6):e0000572. doi: 10.1371/journal.pgph.0000572 (PMC10021283; doi:10.1371/journal.pgph.0000572)
Supplement: Attachment — Submitted filename: Response reviewers vaccine wastage.docx [file pgph.0000572.s003.docx]

Dear Editor

Thank you for considering our study for publication in your journal. Please see below responses to your comments and that of the reviewers. The changes have been highlighted in the manuscript.

Journal Requirements:

1. Please amend your detailed Financial Disclosure statement. This is published with the article, therefore should be completed in full sentences and contain the exact wording you wish to be published.

State what role the funders took in the study. If the funders had no role in your study, please state: “The funders had no role in study design, data collection and analysis, decision to publish, or preparation of the manuscript.”

**Response:** We have amended the financial statement on page 35

2. Please update your Competing Interests statement. If you have no competing interests to declare, please state: “The authors have declared that no competing interests exist.”

**Response:** This has been added on page 35

3. In the online submission form, you indicated that “The data will be shared upon request.”. All PLOS journals now require all data underlying the findings described in their manuscript to be freely available to other researchers, either 1. In a public repository, 2. Within the manuscript itself, or 3. Uploaded as supplementary information.
This policy applies to all data except where public deposition would breach compliance with the protocol approved by your research ethics board. If your data cannot be made publicly available for ethical or legal reasons (e.g., public availability would compromise patient privacy), please explain your reasons by return email and your exemption request will be escalated to the editor for approval. Your exemption request will be handled independently and will not hold up the peer review process, but will need to be resolved should your manuscript be accepted for publication. One of the Editorial team will then be in touch if there are any issues.

**Response:** We have uploaded the study data not shown in the text as supplementary information**.**

4. We ask that a manuscript source file is provided at Revision. Please upload your manuscript file as a .doc, .docx, .rtf or .tex. If you are providing a .tex file, please upload it under the item type ‘LaTeX Source File’ and leave your .pdf version as the item type ‘Manuscript’.

**Response:** The manuscript has been uploaded as a word document.

5. Please provide separate figure files in .tif or .eps format only and remove any figures embedded in your manuscript file. Please ensure that all files are under our size limit of 20MB.

For more information about how to convert your figure files please see our guidelines: https://journals.plos.org/globalpublichealth/s/figures

**Response:** We have uploaded separate figure files in .tif format

6. We notice that your supplementary tables are included in the manuscript file. Please remove them and upload them with the file type 'Supporting Information'. Please ensure that all Supporting Information files are included correctly and that each one has a legend listed in the manuscript after the references list.

**Response:** This has been done. Thank you

Additional Editor Comments (if provided):

The manuscript is within scope of the journal and is of immense significance not only in the geographic area of focus but also overall in the domain - considering the topic at hand. In addition to comments raised by reviewers, authors might need to address following:

Abstract:
**Comment**: In methods section information on how health facilities were selected might be added. Any implication of the sampling on the results might also be highlighted in the conclusion.

**Response:** The first sentence in the methods of the abstract has been modified to read ‘*Twenty-two health facilities were selected randomly from six purposefully identified provinces in the Solomon Islands and across the different levels of the health service.’* (Lines 20-22)

Main text:
**Comment:** Please remove funding information from the aim of the project. State the aim only (line 62-64)

**Response:** The name of the funder has been removed from the main text.

**Comment**: The heading of country background might be replaced by setting and merged with the heading of EPI. This can be more concise.

**Response:** Thank you for your suggestion, we have revised the heading accordingly.

**Comment**: In sampling, please clarify how SLMS were selected? Was it also purposive? Was it based on convenience? This would also feed into discussion and the consequent conclusions derived from the data.

**Response:** The selection of the provinces was purposeful. The other facilities including the SLMs however were selected proportionate to the total number of facilities in the province.

We stated in line 105-108 that ‘*The provinces were purposefully selected to include the two largest, two smallest and Honiara which hosts the national medical store. Within each Province, SLMS, AHCs and RCHs and NAPs were randomly selected with the total number proportionate to the number of facilities in the Province.’*

In the discussion we had mentioned the limitation of our choice of provinces but also pointed to the advantage of having a wide range of facilities. Lines 377-380.

*‘We purposefully selected the provinces and showed very little data from the two remotest provinces of Temotu and Renbel. The other provinces may be different and hence we cannot generalise our results. By selecting the closest and the farthest we have to a large extent provide a good overview of the challenges in the islands and since the three provinces with data account for over 60% of the vaccine consumption, the estimates are likely to be close to the national rates. ‘*

**Comment:** Data sources should precede Data collection section

**Response:** Thank you, we have revised the sections as suggested.

**Comment:** Though not directly relevant from the data, authors might want to add a few sentences on what the results would mean considering the pandemic and expected impact of climate change in Solomon Islands (data is pre-pandemic).

**Response:** We appreciate your suggestion and have included the sentences below in the discussions in lines 357-363

*‘Conversely the impact of the COVID-19 pandemic which has had immense disruptions on immunisation services globally^[1]^ posing additional threat to already weak systems. The Solomon Islands are further challenged by climate change. A recent study in the Pacific, showed that >70% (219/303) of the health facilities in Solomon Island were in locations highly susceptible to sea level rise, particularly during high tides and storm surges^[2]^. Moving forward, strengthening health information systems will be crucial for building resilience.’*

Reviewer #1: This is an important study for economic and epidemiological reasons. It highlights difficulties in efficiently maintaining stocks of EPI vaccines, and making them widely available to individuals even though many vaccines are provided in multi-dose vials which can expire. Overall, the study was well conducted and the discussion raises recommendations in line with the study results.

**Comment:** I feel like an additional point to consider from your paper would be to increase vaccine durability. Has much research been done on re-usability of multi-dose vials, etc.?

**Response:** WHO has a policy on the reuse of multidose vial which states that multi dose liquid vaccine vials, from which one or more doses have been administered, may be used for subsequent immunization session up to 28 days in the recommended manufacture storage conditions provided that certain condition must be met. Studies have shown that the introduction of the MDVP policy has contributed to reducing wastage. We referred to one such study in line 326 ^[3]^.

***Comment:*** Line 72: could you check the birth cohort number?

**Response:** Thank you for spotting the error, this has been corrected (line 73)

**Comment:** Can you mention what is a single dose and multi dose vial vaccine in the methods?

**Response:** We have included the following sentence in line 83 *‘i.e. single dose vials containing only one dose or multi-dose vials with more than one dose.’*

**Comment:** As is, Tables 3 and 4 are okay, but they contain a lot of information. I would maybe move that to a supplementary appendix, and then just summarize the ranges of numbers within a table in the main text.

**Response:** We provided the data in the supplementary information with details for each site by year and summaries by province in the main manuscript.

**Comment**: Could you explain somewhere what a negative number would mean? (perhaps a limitation of problems with bookkeeping, or getting vaccines transferred between clinics?)

**Response:** The negative numbers are doses unaccounted for at the time of physical counting buttressing the challenges with the recording. We considered these to be wasted, missing or damaged and included them in the wastage rate estimates. The footnote for table has been revised.

*^c^ Inventory- The inventory covers physical count at the stores; (+ve) are surplus doses and (-ve) are unaccounted for doses. These negative numbers recorded following stock inventory at NMS were included as wasted/discarded/missing vaccines in wastage rate estimation.*

**Comment**: Could you define/ write out VVM?

**Response:** We have defined a vaccine vial monitor (VVM) as a label containing a heat-sensitive material which is placed on a vaccine vial to register cumulative heat exposure over time. (see line 473) The acronym is also written in full at the time of first use in line 253.

**Comment:** In the discussion you mention an "acceptable range" for wastage rates. Could you define what that would be when you first mention it (paragraph 4 you start to get into this - but should move this material forward, or even add to introduction).

**Response:** The concept of acceptable vaccine wastage has been included in the introduction Line 50-53 reads:

*‘Vaccine wastage may be unavoidable but needs to be within acceptable rates. For example, opening a multidose vial for a few children in a rural setting to avoid missed opportunities in a hard-to-reach area is more acceptable than wastage due to freezing or expiry.’*

Reviewer #2: This manuscript presents the vaccine wastage in Solomon Islands. The manuscript gives important information about the vaccine logistics management in Solomon Islands. The manuscript can be improved further if the following points are addressed.

**Comment**: 1. Introduction
Should mention, if there is any practice of monitoring vaccine wastage periodicals as part of the program or it is done as an adhoc manner like this project.

**Response:**  Solomon Islands has a routine vaccine stock monitoring system in place with monthly physical count at the national and some of the second level medical stores at the moment. This is in turn used to update records in the m-Supply logistics management information system and to request vaccine replenishments from the UNICEF regional vaccines buffer storage. Vaccine wastage rates are not routinely estimated and monitored; this study is the first documented attempt to estimate the actual vaccines wastage rates in the country. A key recommendation from the vaccine wastage reduction strategy is for the ministry of health and the national medical stores with support from partners to institutionalize a vaccines wastage surveillance system in the country as part of the wastage reduction strategy. We have included in lines 63-65 the following sentence

*‘Vaccine wastage rates are not routinely estimated and monitored in the country and this study was the first documented attempt to estimate the actual vaccines wastage rates in the country.’*

**Comment:** 2. Methods- should mention
What are the types of cold chain devices used for storage at the different levels and their power supply type? How the program covers the islands for service delivery? How the vaccines are transported between these levels of stores at what frequency?

**Response:** Solomon Islands uses WHO-prequalified active and passive cold chain devices. The active cold chain equipment comprises of solar direct drives (SDDs) refrigerators which are found in most clinics across the country having no mains power supply, compression type ice-lined refrigerators mostly in the urban centers and SLMS connected to the mains power supply, a central vaccine cold room at the national medical stores which is the first point of all vaccine storage in the country, and freezers for storing ice-packs and vaccines needing freezing such as oral polio vaccines (OPV). The country previously had a large number of absorption gas refrigerators which are now almost phased out. The passive cold chain equipment used in the country include WHO-prequalified cold boxes and vaccines carriers, and a few long range passive devices.

Vaccines are delivered from the national medical stores (NMS) to the second level medical stores (SLMS) every two months, including direct delivery to some isolated clinics such as to Pelau in the remote Malaita outer Islands (MOI). Vaccines delivery to the SLMS is by plane in areas with access to flights and by boats in areas without flight access. From the SLMS, vaccines are delivered every month to the frontline clinics or weekly for the child welfare clinics in areas without functional active cold chain on site. Government funds are used to cover vaccine delivery costs across the supply chain with support from partners.

To explain further, we have included the following paragraph in the methods section (Lines 93-100)

*‘Delivery from the central stores to the SLMs and directly to isolated clinics is bi-monthly, by air or by boat for areas without flight access. From the SLMS, vaccines are delivered every month to the frontline clinics or weekly for the child welfare clinics in areas without functional active cold chain on site. Vaccines are stored in solar refrigerators which are found in most clinics across the country that have no mains power supply, or compression type ice-lined refrigerators mostly in the SLMs connected to mains power supply. SLMs also have freezers for storing ice-packs and vaccines needing freezing. Cold boxes and vaccines carriers are available.’*

**Comment:** in data collection: better to specify the months: Jan-Dec for the years

**Response:** Done (line 131)

3. Results
**Comment:** 3.1. Fig 2: It shall be better to give the number for Guadalcanal as others.

**Response:** In Guadalcanal province the facilities receive their supplies from the Guaducanal store which is different for the other provinces where there is no store and facilities receive supplies from SLMs. Figure 2 has been revised, with the names of all Provinces now stated in the figure rather than as a footnote.

**Comment:** 3.2. Table 1: What does +ve and -ve indicate: please clarify in foot note.

**Response**: The positive numbers are surplus doses at the time of physical counting and the negative numbers are those unaccounted for. These show the challenge in the documentation process. We have revised the footnote in table 1 to read (lines 210-211):

*^c^ Inventory- The inventory covers physical count at the stores; (+ve) are surplus doses and (-ve) are unaccounted for doses. These negative numbers recorded following stock inventory at NMS were included as wasted/discarded/missing vaccines in wastage rate estimation.*

**Comment:** Can mention the damaged doses also here as a column.

**Response**: We do not have the specific information on damaged doses. We got reports of expired doses and these are shown on the table. We have clarified further under limitation the need for more specific data on type of wastage.

**Comment:** 3.3. Table 2: It may be better to add the number of vaccines received/supplied also.

**Response**: These are shown in the supplementary information. We limited table 2 to the number immunised and used these to estimate the wastage rates. There was more missing data for the number of vaccines received/supplied.

**Comment:** 3.4. Overall wastage rates
How the 100% wastage rates were explained- no session held or all vials were damaged?

**Response:** While the reasons put forward are possible, we cannot say for sure as the data were incomplete**.** We clarified in the footnote that for 100% wastage, wasted doses were recorded however the immunisation records were missing. See line 260. We additionally stated in line 314/315 that we did not include the size of the immunisation clinics. This could have given more insight on vaccine usage.

**Comment:** 3.5. Types of wastage
Were the differences in stock consistent across the years and for all types of vaccines or periodic observations?

**Response:** At the time of the survey, stock outs were reported ranging from two facilities not having PCV, TT, OPV and BCG to five facilities not having Hep B. We noted that all facilities had Penta. Assessing the physical stock at the time of the visit though limited does give an indication of the challenges with only one vaccine present in all facilities surveyed. Data on available stock over the period were not available in several facilities. The challenge with documentation has been highlighted and we include a call for more complete data in line 362/363 and strengthening the entre health information system in line 374.

3.6. Fig 3:
**Comment:** What does the VVM mean- due to VVM expiry?

**Response:** We stated VVM stage 3/4. Once the VVM gets to this stage the vial is recommended not to be used. VVM – vaccine vial monitor is now defined (Line 253) and a definition given in line 472-473

**Comment:** what dose wastage mean- date expiry?

What does the wastage mean- due to opened vial wastage?

**Response:** This is one of the limitations, because we are not able to tell the type of wastage. These doses were only reported as wasted. More specific details should be reported. We have included the following sentence in the limitations (lines 371-374).

*‘Our results on the type of vaccine wastage are limited with close to half of the documented doses simply recorded as wasted or damaged which is not specific enough. To be useful for decision making, more detailed reports on the reason for wastage are needed.’*

3.7. Narration for Table 5
**Comment:** How were the vaccines stored in absence of the refrigerators? Were the VVM related wastage could be linked to the periods of no refrigerator?

**Response:** In the absence of refrigerators, vaccines are stored at a nearby facility and transported on clinic days. We have included a paragraph on the type of storage and mode of transportation in lines 93 to 100.

It is possible that the VVM is related to challenges with refrigeration, but we are not able to tell. We had stated in the footnote for figure 3 that ‘*Power outage/off and refrigeration working function well were reported but the number of doses discarded for these reasons was not stated and hence not reflected in the proportions on the chart.’* Lines 469-470.

3.8. Vaccines - Physical count at the facilities
**Comment:** What was the level or agreement and/or differences between the stock numbers from the record/register and physical count?

**Response**: We stated that only four facilities had up to date records. We have moved the sentence to the paragraph on Physical count and clarified further. It now reads

*‘Only four facilities (20%) had up to date records of their stock in the record books.* *This was confirmed during physical counting at the time of the visit’.* (Lines 286-287)

3.9. Table 5
**Comment**: Section- Vaccines returned to cold chain
Which vaccines- used, unused or partially used? - need to clarify

**Response**: We are referring to vaccines that meet the Multidose vial policy which states that multi dose liquid vaccine vials, from which one or more doses have been administered, may be used for subsequent immunization session up to 28 days in the recommended manufacture storage conditions provided that certain condition must be met. They should be well labelled and stored in appropriate conditions.

We have added the following sentence in as a footnote; ^‘^*refers to vaccines for which the multidose vial policy applies i.e those that can be reused up to 28 days after opening if appropriate conditions apply.’* (Lines 292-293)

4. Discussion
**Comment:** 4.1. Should discuss the potential reasons and factors influencing the high wastage in some islands and for some vaccines than others. Should discuss the vaccine record keeping status and need for improvement with appropriate documentation for wastage estimation.

**Response**: We discussed the reasons for the differences in wastage from multidose vials and single dose vials. Regarding differences between islands, access, and population are among the challenges. We did not collect data on the size of the immunisation clinics, 15 facilities did not have the target population displayed. For those with the information, there was a wide variation for target under 1 year of age population; as low as 4 and as high as 1000 in facilities. We stated in lines 314-316.

‘*We did not assess the size of the immunisation clinics, but several facilities recorded less than 10 doses of BCG for a whole year. More complete data are needed to understand the extent of the differences.’*

The need for proper documentation has been highlighted in the above sentence in line 316 and the sentences below in lines 371-374.

*‘Our results on the type of vaccine wastage are limited with close to half of the documented doses simply recorded as wasted or damaged which is not specific enough. To be useful for decision making, more detailed reports on the reason for wastage are needed.’*

**Comment:** 4.2. Limitations should mention the lack of information about the type of wastage in some islands.

**Response**: The following sentence has been added to lines 371-374

*Our results on the type of vaccine wastage are limited with close to half of the documented doses simply recorded as wasted or damaged which is not specific enough. To be useful for decision making, more detailed reports on the reason for wastage are needed.*

5. Supplementary table 2
**Comment:** Can add population size also in the table.

**Response:** The table has been revised to include the population

1. Shet A, Carr K, Danovaro-Holliday MC, Sodha SV, Prosperi C, Wunderlich J, et al. Impact of the SARS-CoV-2 pandemic on routine immunisation services: evidence of disruption and recovery from 170 countries and territories. Lancet Glob Health. 2022;10(2):e186-e94.
2. Taylor S. The Vulnerability of Health Infrastructure to the Impacts of Climate Change and Sea Level Rise in Small Island Countries in the South Pacific. Health Serv Insights. 2021;14:11786329211020857.
3. Patel PB, Rana JJ, Jangid SG, Bavarva NR, Patel MJ, Bansal RK. Vaccine Wastage Assessment After Introduction of Open Vial Policy in Surat Municipal Corporation Area of India. Int J Health Policy Manag. 2015;5(4):233-6.
